# Virtual Screening and Hit Selection of Natural Compounds as Acetylcholinesterase Inhibitors

**DOI:** 10.3390/molecules27103139

**Published:** 2022-05-13

**Authors:** Mariyana Atanasova, Ivan Dimitrov, Stefan Ivanov, Borislav Georgiev, Strahil Berkov, Dimitrina Zheleva-Dimitrova, Irini Doytchinova

**Affiliations:** 1 Chemistry Department, Faculty of Pharmacy, Medical University of Sofia, 1000 Sofia, Bulgaria; idimitrov@pharmfac.mu-sofia.bg (I.D.); stefan@redesignscience.com (S.I.); dzheleva@pharmfac.mu-sofia.bg (D.Z.-D.); idoytchinova@pharmfac.mu-sofia.bg (I.D.); 2 Redesign Science, 180 Varick St, New York, NY 10014, USA; 3 Institute of Biodiversity and Ecosystem Research, Bulgarian Academy of Sciences, 1113 Sofia, Bulgaria; bobogeorgiev5@gmail.com (B.G.); berkov_str@yahoo.com (S.B.)

**Keywords:** acetylcholinesterase (AChE), natural compounds, virtual screening, molecular docking, molecular dynamins, Alzheimer’s disease (AD)

## Abstract

Acetylcholinesterase (AChE) is one of the classical targets in the treatment of Alzheimer’s disease (AD). Inhibition of AChE slows down the hydrolysis of acetycholine and increases choline levels, improving the cognitive function. The achieved success of plant-based natural drugs acting as AChE inhibitors, such as galantamine (GAL) from Galanthus genus and huperzine A from Huperzia serrate (approved drug in China), in the treatment of AD, and the fact that natural compounds (NCs) are considered as safer and less toxic compared to synthetic drugs, led us to screen the available NCs (almost 150,000) in the ZINC12 database for AChE inhibitory activity. The compounds were screened virtually by molecular docking, filtered for suitable ADME properties, and 32 ligands from 23 structural groups were selected. The stability of the complexes was estimated via 1 μs molecular dynamics simulation. Ten compounds formed stable complexes with the enzyme and had a vendor and a reasonable price per mg. They were tested for AChE inhibitory and antioxidant activity. Five compounds showed weak AChE inhibition and three of them exhibited high antioxidant activity.

## 1. Introduction

Neurodegenerative diseases are characterized by the progressive and irreversible loss of neurons from specific regions in the brain. Alzheimer’s disease (AD) is associated with a degeneration of hippocampal and cortical neurons due to the overproduction of cytotoxic β-amyloid (Aβ) peptides followed by an extracellular formation of plaques and intracellular deposition of hyperphosphorylated tau (τ) protein-forming neurofibrillary tangles (NFTs) [1,2]. Neuronal death leads to a reduction in acetylcholine (ACh) levels in synaptic clefts expressed by short-term memory loss and impairment in thinking, speaking, learning, orientation, judgment, and communication [1,3,4]. Cognitive dysfunction is related to two biochemical factors—continuous loss of neurotransmitter ACh due to hyperactivity of the enzyme acetylcholinesterase (AChE) responsible for breaking down ACh into choline, and hyperactive N-methyl-D-aspartate (NMDA) glutamate receptors [5]. One of the therapeutic approaches to AD is the inhibition of AChE, leading to an increase in ACh levels and amelioration of the cognitive function.

The binding site (BS) of AChE is well studied. It is a deep and narrow gorge consisting of several domains: catalytic, anionic, acyl, oxyanion, and peripheral anionic [6,7,8,9,10,11]. The most important among them are catalytic anionic site (CAS) at the bottom of the BS and the peripheral anionic site (PAS) at the entrance and the gorge. PAS is responsible for the initial recognition of the positively charged substrate by Tyr72, Asp74, Tyr124, Trp286, and Tyr341. PAS also is capable of allosterically modulating the activity of the catalytic surface [12]. Additionally, PAS is involved in non-cholinergic functions such as amyloidosis [13], neurite outgrowth, and cell adhesion [14,15]. The substrate trapped by PAS is guided by the other domains to reach CAS. The selective binding to ACh is determined by the acyl subsite consisting of the bulky residues Phe295 and Phe297. The quaternary trimethylammonium choline moiety of ACh forms cation–π interactions with the aromatic residues of the anionic domain—Trp86, Tyr133, Tyr337, and Phe338. In the oxyanion part, constructed by Gly121, Gly122, and Ala204, the substrate tetrahedral transition state is stabilized by a hydrogen-bond network formed between the adopted structural water molecule and the enzyme. The catalytic triad—Ser203, Glu334, and His447 of CAS performs the hydrolysis of ACh by the double-displacement mechanism [16,17].

The AChE inhibitors (AChEIs) marketed currently include donepezil (trade name Aricept), rivastigmine (trade name Exelon), galantamine (GAL) (trade name Reminyl and Nivalin), and tacrine [18]. The first approved drugs for AD treatment tacrine and donepezil have synthetic origin. Tacrine is rarely prescribed due to its hepatotoxicity [19,20,21] and contentious efficacy [22]. Rivastigmine, approved by the FDA in 2000, is designed from physostigmine—a plant alkaloid isolated from *Physostigma venenosum* [23]. In 2001, GAL, an alkaloid from *Galanthus* genus, was approved for AD treatment [24]. Huperzine A, an alkaloid from *Huperzia serrate*, is used as a dietary supplement for memory function improvement in the USA and has been approved for AD treatment in China [25,26].

Natural compounds (NCs) are often assumed to have better tolerance and safety than synthetic molecules, although side effects including allergic reactions, toxicity, and interactions with drugs have been reported for many products [27,28]. Two of the five currently approved drugs for AD treatment have natural origin. Additionally, there are several extensive reviews on natural AChEIs originating from plants, marine organisms, and fungi [29,30,31,32,33,34]. The major groups of compounds with AChE inhibitory activity are alkaloids, coumarines, flavonoids, stilbenes, and terpenoids [29,30,31,32,33,34,35,36].

Computer-aided drug design is a powerful technique for the design of new drugs. Structure-based design, including molecular docking (MD), virtual screening (VS) and molecular dynamics simulations (MDS), is widely used for deep understanding of the intimate mechanisms of ligand–protein interactions, discovering hits and leads from massive databases and design of new ligands. Docking studies of small group plant-based alkaloids on AChE revealed pleiocarpine as the most promising and potent AChE antagonist [37]. Similarly, 100 terpenoids collected from different sources were analyzed for their inhibitory activity on AChE and amyloid beta peptide via docking and MDS [38]. The powerful effect of VS by MD of NCs databases on different targets, including DNA, was reviewed by Ma et al. [39]. Ambure et al. [40] performed a pharmacophore-based VS followed by MD analyses on InterBioScreen’s Natural Compound database in order to discover novel AChEIs [40]. The initial dataset of 47,868 NCs (available in 2012) was screened by multiple filters and a final set of 12 molecules was selected as potential AChEIs on the basis of their interaction patterns and docking scores.

To the best of our knowledge, there is no comprehensive study on all available data-bases of NCs for potential AChE inhibitory activity via molecular docking. Here, we report an MD-based VS of 11 ZINC12 databases of NCs (~150,000 compounds) for AChE affinity. The compounds were subsequently screened for blood brain barrier (BBB) permeability, gastrointestinal (GI) absorption, drug-likeness, lead-likeness, and PAINS (Pan Assay INterference Structures) by SwissADME online server tool [41]. Next, the eligible compounds were visually inspected according to their size and position within the binding pocket. MDSs were performed on the hit compounds in order to evaluate the stability of the complexes. The final set of 10 selected hits was tested experimentally.

## 2. Results

### 2.1. Virtual Screening by Molecular Docking

Almost 150,000 NCs were subject to VS based on the crystallographic structure of human-recombinant AChE (*rh*AChE, pdb id: 4EY6, R = 2.40 Å) [6]. The NCs were collected from 11 databases freely available on the ZINC12 platform: AfroDb_NP, AnalytiCon_DB, HerbalIngredients_DB, HIT_DB, IBScreen_NP, Indofine_NP, NPACT_DB, Nubbe_NP, Specs_NP, TCM_DB_Taiwan, and UEFS_NP. The docking protocol is described in Materials and Methods. The pose with the highest ChemPLP score out of 100 runs was considered for each ligand. Only compounds with docking score equal to or higher than 70 were considered as AChE binders. This cutoff value was chosen to be close to that derived for GAL (72.11) with an RMSD of 0.215 Å.

### 2.2. ADME Filtering and Visual Inspection

**Figure 1:**
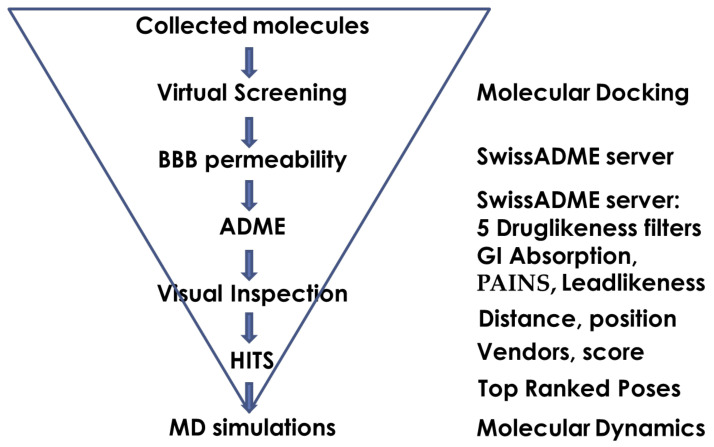
Hit selection by docking-based VS and ADME filtering.

A three-step filtering was applied for hit selection (Figure 1). The first filter was blood—brain barrier (BBB) permeability, a necessary requirement for activity in the brain. It was assessed by the SwissADME server [41]. The number of ligands that passed this step was 40,613. In the next step, eight additional SwissADME filters were applied: five for drug-likeness (rules of Lipinski, Ghose, Veber, Egan and Muegge), one for gastrointestinal (GI) absorption, one for PAINS allerts, and one for lead likeness. Only 8315 of NCs fulfilled all eight criteria. At the last step, a visual inspection of the binding poses within the protein binding site was performed along with measuring the size of the aromatic moiety occupying the CAS. Only ligands bound near the PAS or deeply in the CAS with aromatic rings sized at least 5 Å in diameter between two terminal heavy atoms were selected. Thus, at the end of this three-step filtering, only 215 molecules remained. The compounds were checked for available vendors, and 32 non-GAL type molecules from 23 structural groups were selected for further analysis (Table S1). They were numbered from 1 to 34, including two GAL derivatives (**10** and **11**).

### 2.3. Molecular Dynamics Simulatioins and Trajectory Analyses

The stability of the complexes formed between the 32 hits and the AChE was tested by 1 μs molecular dynamics simulation. Initially, the stability of the complexes was inspected visually. In 11 complexes, the ligands almost exited the BS (3, 4, 6, 24, 27, and 30) or occupied an allosteric site, and CAS remained accessible (8, 12–15). In the rest of the 21 complexes, the ligands remained in the BS during the whole simulation with ligand RMSD < 1.5 Å and protein RMSD < 2.5 Å. Five of the ligands had RMSD up to 0.5 Å (16, 31–34), for 10 of them the RMSDs varied between 0.5 and 1 Å (1, 7, 18, 19, 20, 22, 25, 26, 28, 29), and 6 molecules had RMSDs between 1 and 1.5 Å (2, 5, 9, 17, 21, 23). The protein Cα RMSDs remained below 2.5 Å, confirming the stability of the systems (Figure S1). The protein Cα RMSFs (root-mean-square fluctuations) showed that the most dynamic residues belong to the unstructured regions on the surface between the 76th and 84th and 384th and 388th positions, around the capped chain breaks at 255th and 491th positions, and to both termini (Figure S1).

The convergence of MM-GBSA energies (ΔH) was assessed over the production phase (Figure S2). In the absence of bridging solvent molecules [42], the enthalpies converged within 100 ns and ranged between −25 and −55 kcal/mol (Figure S2). In the presence of solvent molecules, the convergence of ΔH_solv_ took longer than 100 ns and had values ranging between −30 and −66 kcal/mol (Figure S2).

Three criteria were applied in the final selection of hits for experimental testing among the 21 NCs: sum of ChemPLP score and the module of ΔH_solv_, single vendor, and price for 1 mg (Table S2). The final set of 10 NCs selected for anti-AChE testing is given in Table 1.

### 2.4. AChE Inhibitory Activity

**Figure 2:**
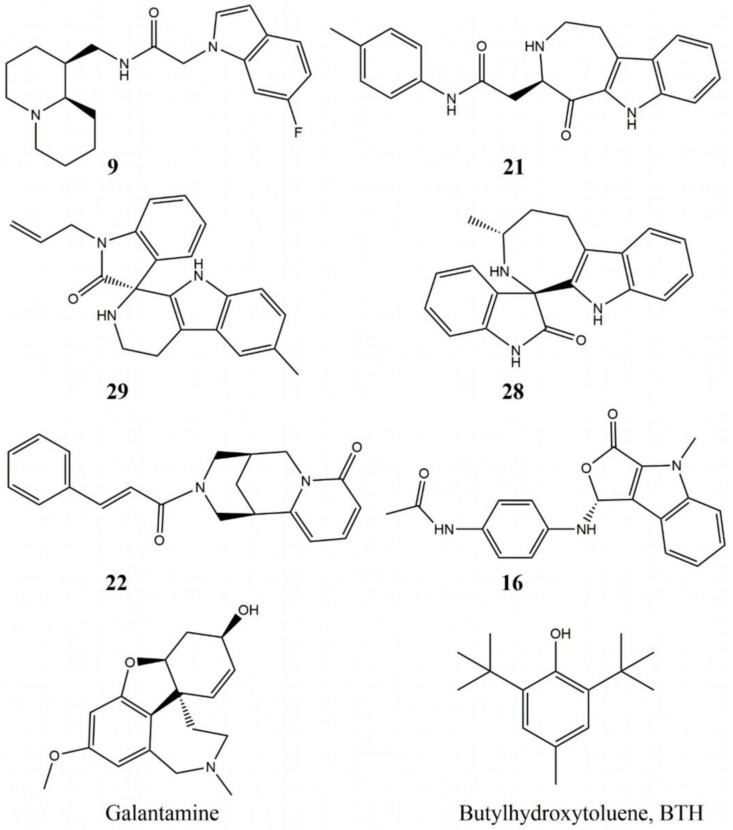
Structures of the novel AChE inhibitors, discovered in the present study: **9** (N-{[(1S,9aR)-octahydro-1H-quinolizin-1-yl]methyl}-2-(6-fluoro-1H-indol-1-yl)acetamide), **21** (N-(4-methylphenyl)-2-{5-oxo-1H,2H,3H,4H,5H,6H-azepino [4,5-b]indol-4-yl} acetamide), **29** (6′-methyl-1-(prop-2-en-1-yl)-1,2,2′,3′,4′,9′-hexahydrospiro[indole-3,1′-pyrido [3,4-b]indol]-2-one), **28** (3-methyl-1′,2′,3,4,5,10-hexahydro-2H-spiro[azepino [3,4-b] indole-1,3′-indol]-2′-one), **22** ((1S,9R)-11-[(2E)-3-phenylprop-2-enoyl]-7,11-diazatricyc lotrideca-2,4-dien-6-one), **16** ((R)-N-(4-((4-methyl-3-oxo-3,4-dihydro-1H-furo [3,4-b] indol-1-yl) amino)phenyl)acetamide), and the two positive controls —galantamine (GAL) and butylhydroxytoluene (BHT).

The AChE activity of the selected 10 NCs was measured by Ellman’s method as described in Materials and Methods. The IC_50_ values are given in Table 1. Five compounds showed IC_50_ < 10 mM. Their structures are given in Figure 2. Two of them, **22** and **28**, have activity in the micromolar range, another two, **9** and **21**, have activities between 1 and 2 mM, and the fifth, **29**, has an activity above 5 mM. All of them are less active than GAL with IC_50_ = 2 µM.

### 2.5. Antioxidant Activity. ABTS Radical Scavenging Activity

As the anti-AChE activity in vivo is associated with increased oxidative stress [43,44], the antioxidant potential of the selected hits was tested as well as described in Materials and Methods. It is given in Table 1 as % ABTS radical scavenging with BHT as a positive control. Two of the hits—**9** and **21**—showed weak ABTS activity of 6.43% and 34.68%, respectively. Compounds **28** and **29** showed high antioxidant activity (80.94% and 70.55%, resp.), while compound **16** showed significant activity of 95.82% comparable to and even higher than the positive control BHT (92.38%).

## 3. Discussion

About 150,000 NCs available in 11 databases at ZINC12 were subject to virtual screening via molecular docking against AChE. The applied docking protocol was previously optimized and used to predict new AChE inhibitors that were experimentally proven [45,46,47,48,49,50,51]. The compounds were screened for BBB permeability, drug-likeness filters, GI absobtion, PAINS, and lead-likeness. Visual inspection of ligand positions within the binding site of AChE followed by size measurement of the condensed ring moiety located at CAS led to a selection of 215 eligible compounds. From them, 32 non-galanthamine type molecules with the highest ChemPLP docking score and available vendor were selected. The stability of their complexes with AChE was estimated via 1 μs molecular dynamics simulation. Twenty one molecules formed stable complexes, and the ΔH values with and without solvent molecules were calculated. Ten molecules from the same vendor with prices up to USD 100 per 1 mg were selected for experimental testing. Five of them showed anti-AChE activity in the micro- to low millimolar range. They are **9**, **21**, **22**, **28**, and **29**.

**Figure 3:**
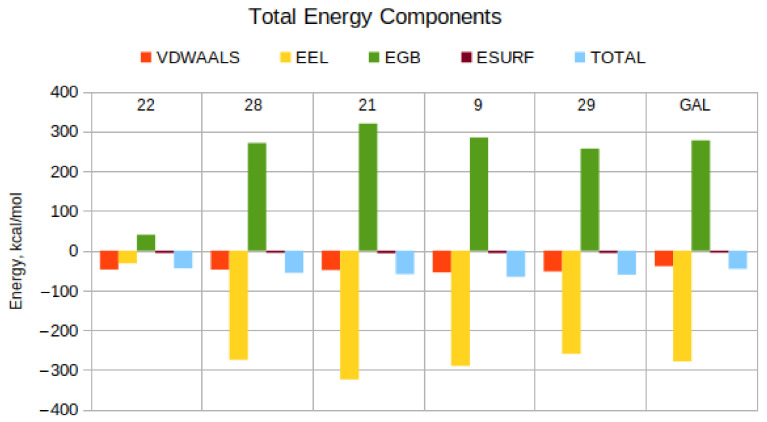
Total energy components of each ligand complexed with AChE. The positive control is GAL. The van der Waals and electrostatic interaction energies are denoted as VDWAALS and EEL, respectively. The polar and non-polar solvation energies are denoted as EGB and ESURF, correspondingly. The total energy of binding is denoted as TOTAL.

In MM-PBSA and MM-GBSA calculations, the magnitude of the resultant ΔH values is sensitive to the choice of atomic radii and nonpolar decomposition scheme [52], whereas the relative energies (relative to each other) are not [42,52]. Thus, while the absolute ΔH values would be different using, for example, bondi and mbondi2 radii, the relative ranking of the compounds by ΔH would be nearly unaffected because all compounds would experience an almost identical amount of error during the calculations. Because the parameters for enthalpy calculations are the same for all complexes, all ΔH values are overestimated by a similar amount, which cancels out in the relative rankings. For example, if compound A scores significantly better than B using bondi radii, it will score better using any other set of atomic radii or decomposition scheme, as long as the same calculation scheme is applied to both compounds. It is precisely this property of relative free energy calculations that enables virtual high-throughput screening. As we aim to screen a large library of compounds, we require a rapidly computable, cheap descriptor of the affinity of the different ligands for the target protein rather than a detailed analysis of the different protein—ligand complexes [53,54,55,56]. The efficacy of our protocol is demonstrated by the high success rate (5 actives out of 10 experimentally tested molecules or 50%), which is orders of magnitude higher than the hit rate of randomly selecting compounds (usually around 0.1–1%) [57]. In Figure 3 and Table 2, we present the energetic components for the studied protein–ligand complexes and briefly discuss them below.

The most active compound **22** is the only one that is not charged at physiological pH. Therefore, its van der Waals (E_VDW_), electrostatic (E_EL_), and polar (E_GB_) solvation values are in a similar range. All other tested compounds, including GAL, have a charge +1 at physiological pH. As expected, their electrostatic and polar solvation contributions are dominant. It is evident, however, that for each compound, these values are very close and almost cancel each other out. Thus, the main contribution to the total binding energy is due to the E_VDW_ term. The non-polar solvation contribution (E_SURF_) is essentially always small and similar for all compounds.

**Figure 4:**
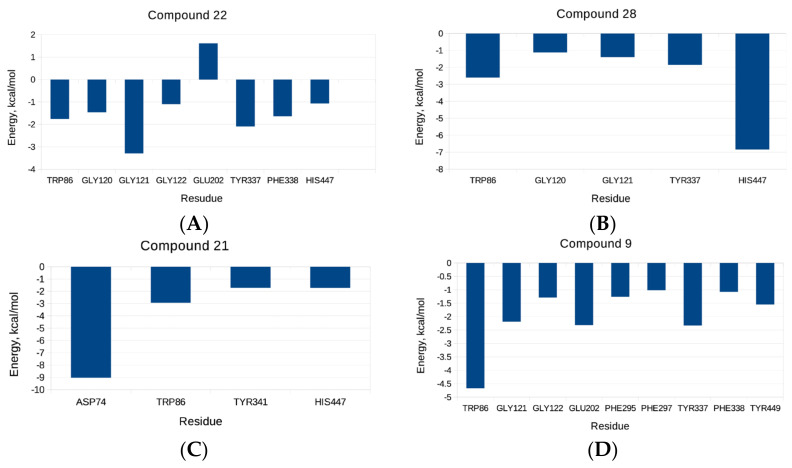

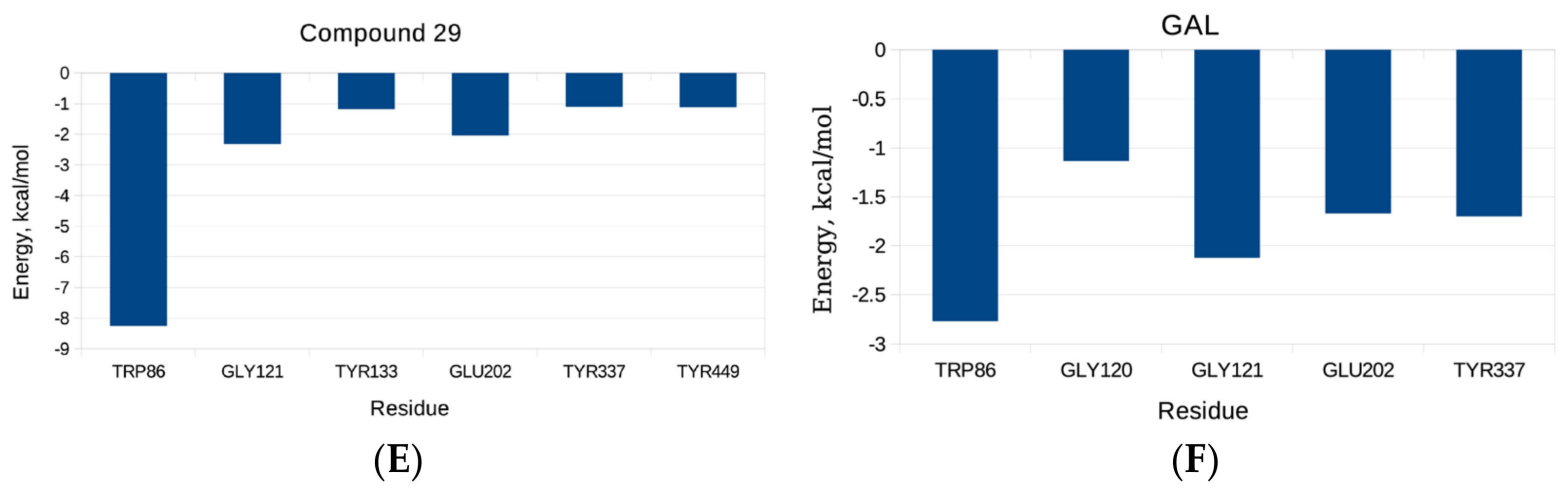
Energy decomposition per residue of predicted compounds (**A**) **22**, (**B**) **28**, (**C**) **21**, (**D**) **9**, (**E**) **29,** and (**F**) GAL. The energy contribution larger than 1 kcal/mol is displayed.

Additional per-residue energy decomposition analysis for the studied compounds was performed, as depicted in Figure 4. It can be seen that in the case of compound **22**, Gly121 from the oxyanion sub-site, followed by Tyr337 from the anionic domain, considerably contributed to the binding energy. Moreover, Trp86 and Phe338 from the anionic sub-site, Gly120 and Gly122 from the oxyanion pocket, and His447 from the CAS contribute to the binding energy. His447 had a significant contribution to the total energy of ligand **28** to AChE, followed by Trp86, Gly120, Gly121, and Tyr337. In the case of compound **21**, a considerable contribution to the binding energy comes from Asp74 from the PAS, followed by Trp86, Tyr341 (PAS), and His447; a sizeable contribution to the binding energy of **9** stems from Trp86, followed by Gly121, Glu202, and Tyr337. The main contributing residue to the total binding energy for compound **29** and the enzyme is Trp86 followed by Gly121 and Glu202. The per-residue energy decomposition for the positive control, GAL, reveals that Trp86, Gly121, Glu202, and Tyr337 are the most highly contributing ones.

**Figure 5:**
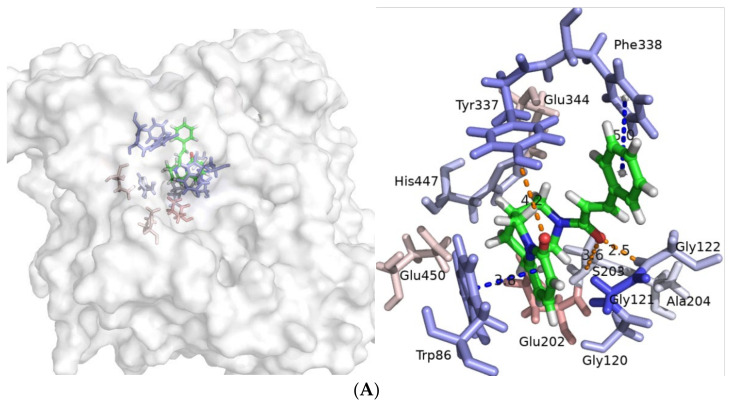

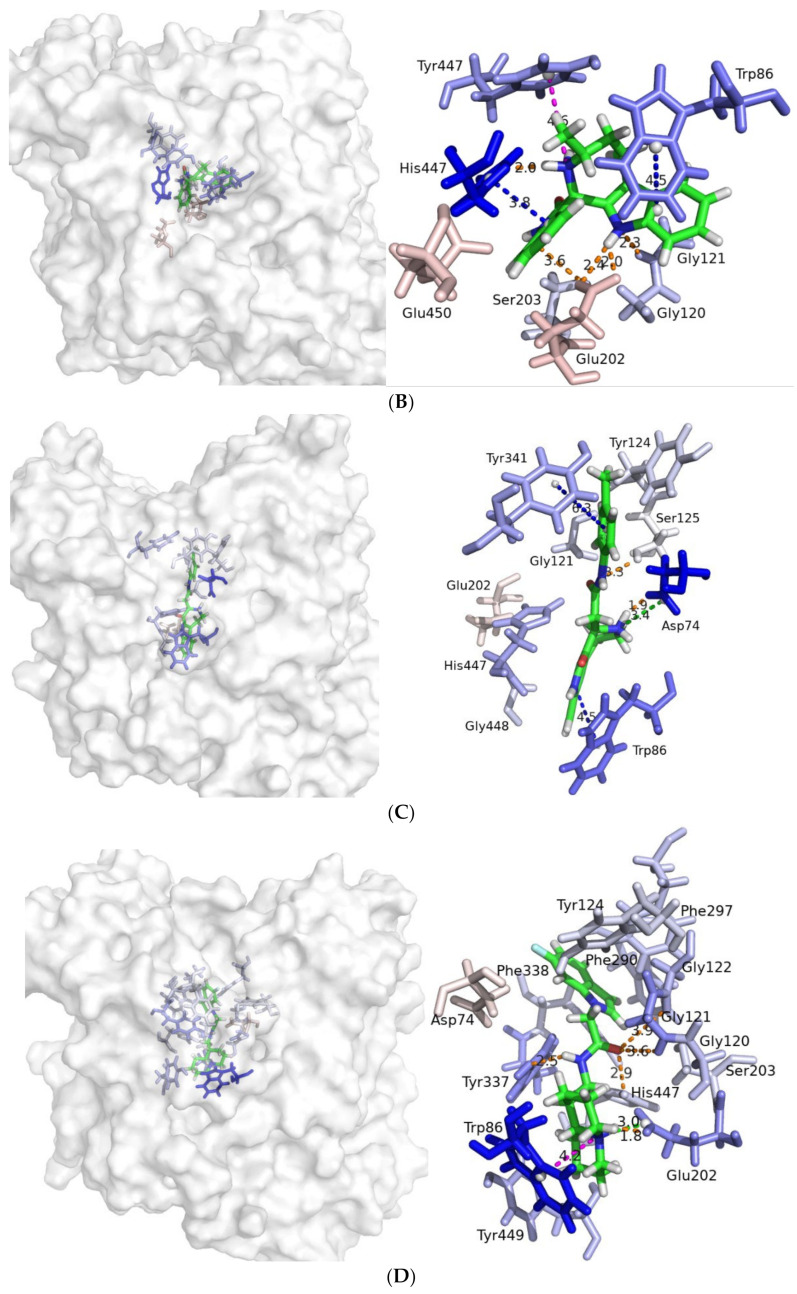

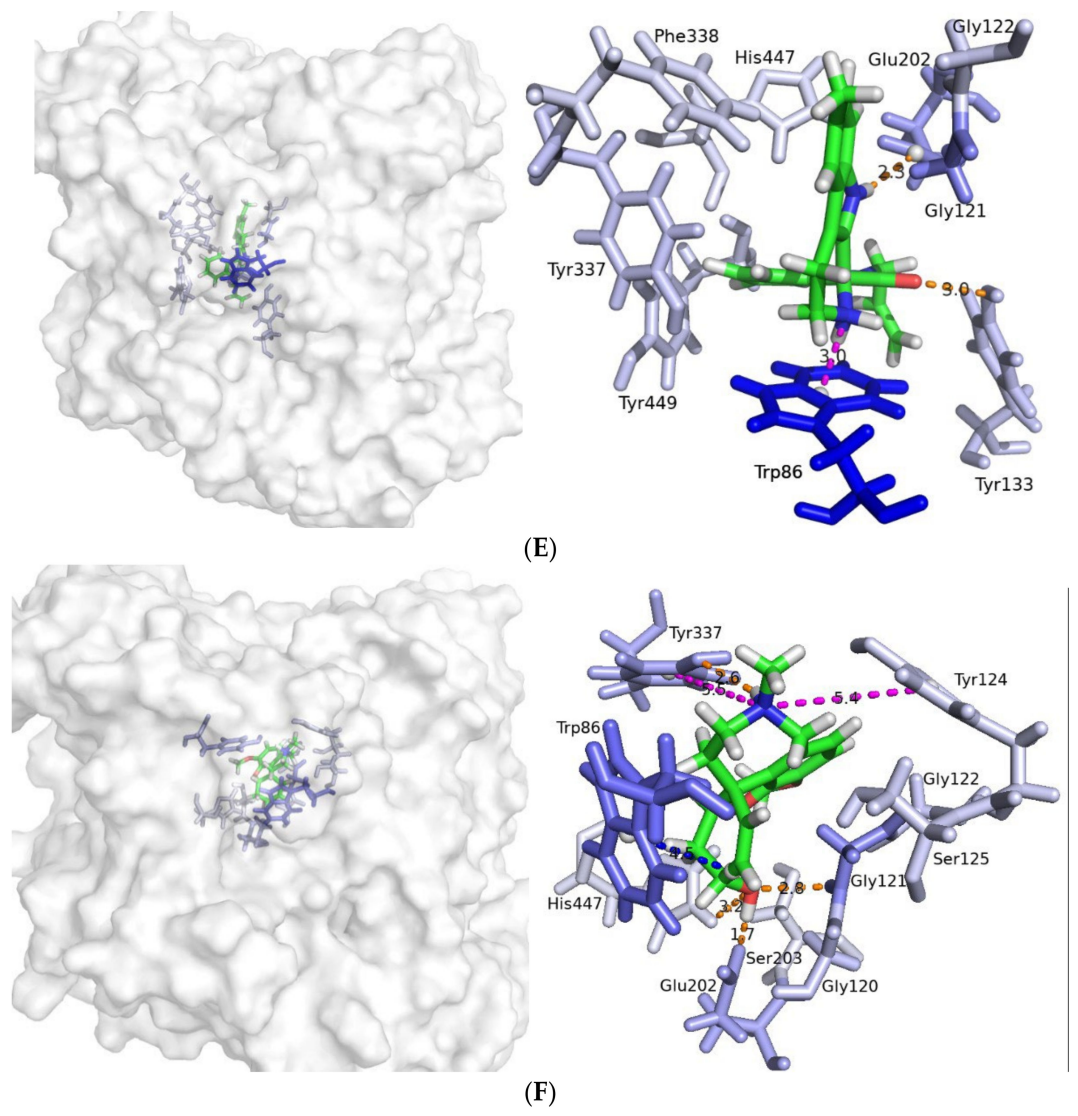
Intermolecular interactions between AChE and compound (**A**) **22** (in 329.8 ns), (**B**) **28** (in 486.4 ns), (**C**) **21** (in 988.4 ns), (**D**) **9** (in 557.6 ns), (**E**) **29** (in 654.8 ns), and (**F**) GAL (364.8 ns). Hydrogen bonds are denoted as orange dashes. Distances between center of mass of π–π or π–p electrons, prerequisite for π–π stacking or π–p contact, are denoted in blue dashes. Cation–π distances are presented in magenta. Salt bridges are denoted with green dashes. Distances are presented in Å. Residues are colored according to their energy contribution—blue colored ones favor the binding, while red colored do not favor it. More saturated color indicates higher contribution. Ligands are colored by element.

To gain further insight into the intermolecular interactions between the ligands and the enzyme during the course of the production, dynamics were analyzed using cpptraj V4.14.0 [58]. Compound **22** forms in three hydrogen bonds. Two of them are formed between the O atom from the carbonyl group of the linker and H atoms at N-terminus of Gly121 and Gly122 (Figure 5A and Figure S3A,B). The third hydrogen bond occurs between the O atom from the carbonyl group from the pyridin-2-one moiety of **22** and H atom from the hydroxyl group of Tyr337 (Figure 5A and Figure S3C). The distance between the pyridin-2-one ring of **22** and indole part of Trp86 is appropriate for a π–π contact (Figure 5A and Figure S3D). Distances between atoms during the course of simulation are shown in Figure S3A–D.

The complex with compound **28** is stabilized via six hydrogen bonds during molecular dynamics. The NH group at the indole moiety acts as a hydrogen bond acceptor and donor with the N-terminus from Gly121 and the carboxyl group from Glu202, respectively (Figure 5B and Figure S4A,B). Similarly, the OH group from Ser203 takes part in two short-lasting hydrogen bonds with NH and CO groups from the indolinone part of **28** (Figure 5B and Figure S4C,D). The N atom at His447 forms hydrogen bonds with both H atoms at the quaternary ammonium group from the ligand (Figure 5B and Figure S4E,F). The distance between the positively charged ammonium atom and center of mas of Tyr337 is a precondition for a cation–π interaction (Figure 5B and Figure S4G). Centers of mass of indole moieties of Trp86 and **28** are at an appropriate distance for π–π contact (Figure 5B and Figure S4H). The distance and position between the centers of mass of His447 and the indolinone part of **28** are a prerequisite for stable π–π stacking observed for a long period during the simulation (Figure 5B and Figure S4I). Distances between atoms during the production dynamics are shown in Figure S4A–I.

A salt bridge is formed during the simulation between the carboxyl group from Asp74 and the quaternary ammonium group from ligand **21** (Figure 5C and Figure S5A,B). One hydrogen bond is formed between the H atom of the side chain hydroxyl group of Ser125 and an O atom from a CO group from the linker (Figure 5C and Figure S5C). Indole moiety from Trp86 is appropriately located for the formation a π–π contact with the indole part from ligand **21** (Figure 5C and Figure S5D). Terminal benzene ring of **21** reaches Tyr341 from PAS, where a π–π interaction can be formed (Figure 5C and Figure S5E). Distance plots in the course of the simulation are presented in Figure S5A–E.

In the case of compound **9**, four hydrogen bonds and one salt bridge stabilize the complex with the enzyme (Figure 5D). A salt bridge is formed between the carboxyl group from Glu202 and the quaternary ammonium group (Figure 5D and Figure S6A,B). Three hydrogen bonds occurred during the simulation between the O atom at the CO group from the ligand’s linker and H atoms at N-terminus of Gly121 and Gly122, and one with H atom from His447 (Figure 5D and Figure S6C,D,F). The oxygen atom of the Tyyr337 hydroxyl takes part in the fourth hydrogen bond with an H atom from the linker amino group (Figure 5D and Figure S6E). The distance between the indole part from Trp86 and the positively charged ammonium atom is appropriate for a cation–π contact (Figure 5D and Figure S6G). Figure S6A–G shows the respective distance plots.

Two hydrogen bonds are formed between compound **29** and the enzyme. The first one is between the hydroxyl hydrogen from Tyr133 and a O atom from indolinone moiety’s carbonyl group. The second one is between carboxyl group from Glu202 and the amino hydrogen from the indole part of the molecule (Figure 5E and Figure S7A,B). The distance between the center of mass of the indole moiety from Trp86 is appropriate for a cation–π interaction with the positively charged ammonium atom of ligand **29** (Figure 5E and Figure S7C). Distance plots are presented in Figure S7A–C.

Additionally for comparison, we analyzed the interactions between the positive control GAL and AChE (Figure 5F). Four hydrogen bonds stabilize the complex between both partners. In three of them, O atom from GAL’s OH group takes part with Gly121, Glu202, and His447, respectively (Figure 5F and Figure S8A–C). The fourth one is formed between the O atom at OH from Tyr337 and the hydrogen atom in the quaternary ammonium group (Figure 5F and Figure S8D). The centers of mass of the benzene ring from Tyr124 and Tyr337 are at appropriate distances for cation–π contacts (Figure 5F and Figure S8E,F). The distance between the center of mass of the indole moiety from Trp86 and the double bond from GAL is a prerequisite for π–π interaction (Figure 5F and Figure S8G). For all of the discussed ligands, multiple steric interactions stabilize the complexes during simulation.

Compounds **9** and **22** are quinolizidine-type alkaloids. Compound **9** is a lupinine derivative containing terminal indole moiety, while compound **22** is a N-substituted cytisine derivative with terminal phenyl ring. It has been shown that lupinine and its tetramethylammonium derivative are reversible inhibitors of AChE [59]. Cytisine is used in smoking cessation [60], but it does not inhibit AChE and BChE [61]. Additionally, quinolizidine alkaloids are known to bind to nicotinic and muscarinic acetylcholine receptors (nAChR and mAChR), as lupinine binds preferably to mAChR, while cytisine binds preferably to nAChR [62]. Hupersine A, isolated from *Huperzia serrata,* is a quinolizidine-type alkaloid acting as a reversible AChE inhibitor and NMDA receptor antagonist [63,64,65,66].

Compound **29** is a dicorynamine derivative. Dicorynamine is a β-carboline alkaloid isolated from *Dicorynia guianensis* Amsh heartwood [67]. It has been found that the crude extract of alkaloids demonstrates the maximum scavenging activity in the ABTS model with IC_50_ = 90.07 µg/mL compared to the positive control ascorbic acid (IC_50_ = 105.90 µg/mL) [67]. In our ABTS study, the dicorynamine derivative, **29**, showed 80.94% ABTS activity, which is close to the positive control BHT, 92.38% (Table 1).

Compounds **21** and **28** are indole-containing compounds. Their structures partially resemble the structure of the indole alkaloid ibogaine found in *Tabernaemontana* species of the Apocynaceae family. Ibogaine has been extensively studied for anti-addictive properties, but clinical trials failed due to cardiotoxicity [68]. Additionally, ibogain-type alkaloids like catharanthine and coronaridine congeners have been found to selectively inhibit the nAChRs and Cav2.2 channels, and to potentiate GABA A receptors [69,70,71,72]. Ibogaine and coronaridine are NMDA receptor antagonists, with K_i_ = 1.1 µM and K_i_ = 6.2 µM, respectively [73].

## 4. Materials and Methods

### 4.1. Virtual Screening by Molecular Docking

Approximately 150,000 natural compounds collected from 11 freely available databases in ZINC12 were screened virtually by molecular docking. The structures were docked into the X-ray structure of recombinant human acetylcholinesterase (rhAChE, pdb id: 4EY6, R = 2.40 Å) [6]. The docking simulations were performed with GOLD v.5.2.2 [CCDC Ltd., Cambridge, UK] using a previously described and optimized-for-this-system protocol [45,46,47,48,49,50,51]. The following settings were used: scoring function ChemPLP, rigid protein, flexible ligand, and a radius of the binding site of 6 Å around the crystallographic structure of GAL. For each ligand, 100 poses were generated. The structural water molecule HOH846B, forming bridging interactions between the crystallographic GAL and enzyme, was excluded from the docking calculations due to the diversity of the screened ligand structures. The highest scored pose for each ligand was considered. The protocol was validated as the crystallographic structure of GAL was removed from the complex and redocked again at the described settings.

### 4.2. ADME Filters

The tested compounds were screened by several ADME filters. The BOILED-Egg (Brain Or Intestinal EstimateD permeation) method based on the lipophilicity and polarity of small molecules [74] implemented in the SwissADME server [41] was used to predict the BBB permeability of the ligands. Lipinski’s filter, also known as the ‘rule of five’, is based on experimental and computational approaches to estimate solubility and permeability in drug discovery [75]. A knowledge-based approach for qualitative and quantitative characterization of known drug databases is used in the Ghose’s filter [76]. The Veber’s filter is based on two criteria: number of rotatable bonds and polar surface area, which are in a good correlation with the experimental oral bioavailability data for over 1100 drug candidates [77]. A statistical model for the recognition of passive intestinal absorption is applied in the Egan’s filter [78]. The Muegge’s criterium presents a pharmacophore point filter using so called “chemical wisdom” that is unbiased from fitting the structural content of specific drug databases to prediction models [79]. The GI absorption prediction is based on the BOILED-Egg method [74]. PAINS alerts help in the identification of frequent hitters or promiscuous compounds in many biochemical high throughput screens based on substructural features [80]. Liead-like combinatorial libraries designed by Teague et al. are applied in the lead-likeness filter [81].

### 4.3. Visual Inspection

The molecules that passed the ADME filters were visually inspected by two criteria: position within the binding gorge, namely if the ligand is placed in the binding site near the PAS or deeply in the CAS; and a size of at least 5 Å for the aromatic moiety was chosen as a requirement to retain the ligand in the binding site. The size was defined as the distance between two terminal heavy atoms of the aromatic ring placed in CAS. The size of the aromatic moiety of GAL in CAS is 6.033 Å.

A mandatory condition for hit selection was the vendor availability and ChemPLP score higher than that of GAL.

### 4.4. Molecular Dynamic Simulations and Trajectory Analyses

#### 4.4.1. System Preparation

The coordinates of pdb id: 4EY6 were used as initial ones for the complex between AChE and GAL [6]. The structural water molecule HOH846B was kept for all MD simulations. As the X-ray AChE structure contains missing regions between residues 258 and 262 as well between 491 and 496, the Ace and NMe caps were added at both ends as well as at both terminal residues of the protein chain. Missing chain regions are far from the ACh binding site on AChE and do not influence the final results. The protein chain was solvated in a truncated octahedral box with TIP3P water [82] as a saline, as the system was kept neutral. The initial coordinates for the selected hit compounds were taken from the docking calculations. Ligand protonation states and charges at physiological pH were used as the pK_a_ values were predicted using ACD/logD tool (Advanced Chemistry Development, Inc., ACD/Labs). Ligand parameters were obtained using the general Amber force field (GAFF 2.11) [83] with AM1-BCC charges [84].

#### 4.4.2. Molecular Dynamic Simulations

The solvated systems were subject to energy minimization, then heated to 300 K for 1 ns, followed by 1 ns of constant pressure density equilibration. Next, the systems were equilibrated for 1 ns under constant temperature (300 K) and pressure (1 bar), using the Langevin thermostat [85] and Berendsen barostat [86], respectively. They were simulated for 1000 ns (1 µs) of production dynamics with the ff14SB force field [87] under periodic boundary conditions. Frames were saved every 200 ps (0.2 ns) for a total of 5000 per trajectory, to be used in subsequent analysis.

#### 4.4.3. Trajectory Processing and MM-GBSA Calculations

The stability of the systems was evaluated via root-mean-square deviations (RMSD) for ligand-heavy atoms, protein carbon alpha (Cα), and root-mean square-fluctuations (RMSF) with respect to the starting coordinates for protein Cα atoms with cpptraj V4.14.0 [58]. For each complex trajectory, the enthalpy of binding between the AChE and the ligand (ΔH) was computed with the MMPBSA.py tool [88], part of the Amber18 package. We used the end-state free energy method, MM-GBSA (molecular mechanics-generalized Born surface area), which includes an implicit solvent analogous to MM-PBSA (molecular mechanics Poisson–Boltzmann surface area) but is computationally cheaper than MM-PBSA [42].

### 4.5. AChE Inhibitory Activity

The compounds selected for testing were purchased from InterBioScreen Ltd.

The microplate assay used for measuring AChE inhibitory activity was performed in 96-well plates using a modified method of Ellman et al., 1961 [89], as described by López et al., 2002 [90]. Acetylthiocholine iodide (ATCI) in solution with 5,5′-dithiobis(2-nitrobenzoic acid) (DTNB) was used as a substrate for the acetylcholinesterase from *Electrophorus electricus* (Sigma-Aldrich, Darmstadt, Germany). All compounds were tested at concentrations from 10^−2^ to 10^−8^ M. Fifty microliters of AChE (0.25 U/mL) dissolved in phosphate buffer (8 mM K_2_HPO_4_, 2.3 mM NaH_2_PO_4_, 0.15 M NaCl, pH 7.5) and 50 μL of the samples dissolved in the same buffer were added to the wells. The plates were incubated for 30 min at room temperature before the addition of 100 μL of the substrate solution (0.04 M Na_2_HPO_4_, 0.2 mM DTNB, 0.24 mM ATCI, pH 7.5). The absorbances were read in a microplate reader (BIOBASE, ELISA-EL10A, China) at 405 nm after 3 min. Enzyme activity was calculated as inhibition percentage compared to an assay using a buffer instead of inhibitor. GAL was used as a positive control. The AChE inhibitory data were then analyzed with the software package Prism 9 (Graph Pad Inc., San Diego, CA, USA). The IC_50_ values were measured in triplicate and the results are presented as means ± SD.

### 4.6. Antioxidant Activity. ABTS Radical Scavenging Activity

2,2′-azinobis-(3-ethylbenzothiazine-6-sulfonic acid) (ABTS), potassium persulphate, and butylhydroxy toluol (BHT) were purchased from Sigma-Aldrich. For ABTS assay, the procedure followed the method of Grochowski et al. [91] with some modifications. The stock solutions included 7 mM ABTS solution and 2.4 mM potassium persulphate solution. The working solution was then prepared by mixing the two stock solutions in equal quantities and allowing them to react for 14 h at room temperature in the dark. The solution was then diluted by mixing 2 mL ABTS solution with 50 mL methanol to obtain an absorbance of 0.705 ± 0.05 units at 734 nm using a Shimatzu 1203 UV-VIS spectropho-tometer (Japan). A fresh ABTS solution was prepared for each assay. One millimeter of each com-pound (125 μL) was allowed to react with 1850 μL of the ABTS solution, and the absorbance was taken at 734 nm after 7 min. The control contains 125 μL methanol and 1850 μL of the ABTS solution. All determinations were performed in triplicate (*n* = 3). The results were expressed as % ABTS activity, using the following equation: % ABTS radical scavenging activity = ((A_control_ − A_sample_)/A_control_) × 100, where A_control_ is the absorbance of the control, while A_sample_ is the absorbance of the sample. Butylhydroxytoluene (BHT) (1 mM in MeOH) was used as a positive control.

## 5. Conclusions

In the present study, five novel naturally originating hits were discovered by multistep virtual screening as promising AChE inhibitors. Two of them (**9** and **22**) are quinolizidine-type alkaloids, another two (**21** and **28**) belong to the indole-type alkaloid family, and one (**29**) is a β-carboline-type alkaloid. Their anti-AChE activities range from 0.39 to 5.7 mM. Additionally, **29** and **28** showed high antioxidant activity. Surprisingly, one powerful an-tioxidant compound was discovered as well. This is compound **16**, which has no AChE activity but possesses antioxidant activity higher than that of the positive control butylhy-droxytoluene. The post-MD simulation analyses reveals the structural features responsible for AChE binding. These are the carbonyl group from the linker and the pyridin-2-one moiety of **22**; the quaternary amonium groups of **9**, **21**, **22**, and **29**; the amide group from the linker; and the quinolizidine moiety of **9**. All together these findings show that the novel molecules are promising hits for further lead optimization and development.

## Figures and Tables

**Table 1:** ChemPLP score, ΔH_solv,avg_, sum of their modules, and anti-AChE and antioxidant activities of the selected NCs are presented. GAL is the reference AChE inhibitor and butylhydroxytoluene (BHT) is the reference antioxidant. The IC50 and ABTS values were measured in triplicate, and the results are presented as means ± SD.

Compund	ChemPLP	ΔH_solv, avrg_, kcal/mol	ChemPLP + |ΔH_solv, avrg_|	IC_50_, mM	ABTS (%)
**5**	85.0500	−30.8296	115.8796	>10	na
**9**	85.9159	−65.8063	151.7222	1.8 ± 0.75	6.43 ± 0.85
**16**	76.7413	−45.8669	122.6082	na	95.82 ± 0.21
**17**	82.7631	−34.3182	117.0813	>10	na
**18**	79.0189	−41.3130	120.3319	na	na
**21**	82.7599	−59.1422	141.9021	1.2 ± 0.19	34.68 ± 1.27
**22**	84.6421	−44.4386	129.0807	0.39 ± 0.16	na
**25**	77.6389	−42.3505	119.9894	Na	na
**28**	73.5909	−55.8388	129.4297	0.62 ± 0.14	70.55 ± 0.85
**29**	77.5505	−60.2032	137.7537	5.7 ± 3.50	80.94 ± 0.94
GAL	72.1100	−45.5914	117.7014	0.002 ± 0.0003	
BHT					92.38 ± 0.21

na—not active.

**Table 2:** Energy components (E_VDW—_van der Waals and E_EL—_electrostatic interaction energies; E_GB—_polar and E_SURF_—non-polar solvation energies solvation energies; TOTAL—total energy of binding) of the ligands complexed with the enzyme. The reference AChE inhibitor is GAL.

Compound	E_VDW_ Kcal/Mol	E_EL_ Kcal/Mol	E_GB_ Kcal/Mol	E_SURF_ Kcal/Mol	TOTAL Kcal/Mol	E_EL +_ E_GB,_ Kcal/Mol
22	−47.15	−31.53	40.20	−5.96	−44.44	8.67
28	−47.40	−274.18	271.14	−5.39	−55.84	−3.05
21	−48.46	−324.22	320.09	−6.55	−59.14	−4.13
9	−54.68	−289.90	284.96	−6.18	−65.81	−4.95
29	−52.21	−259.20	257.11	−5.91	−60.20	−2.08
GAL	−39.18	−278.77	277.42	−5.06	−45.59	−1.35

## Data Availability

Not applicable.

## Supplementary Materials

The following supporting information can be downloaded at: https://www.mdpi.com/article/10.3390/molecules27103139/s1, Figure S1: Ligands heavy atoms (up) and protein backbone C-atoms RMSDs (in the middle) and RMSFs (down) for AChE; Figure S2: Convergence of enthalpies with excluded and included solvent interactions. Figure S3. Distance plots over the course of simulation for compound **22**. (A) between H atom at N-terminal from Gly121 and O atom at CO group from the linker; (B) between H atom at N-terminal from Gly122 and O atom at CO group from the linker; (C) between H atom at OH group from Tyr337 and O atom at CO group from pyridin-2-one; (D) between indole part of Trp86 and pyridin-2-one. Figure S4. Distance plots over the course of simulation for compound **28**. (A) between H atom at N-terminal from Gly121 and N atom from indole moiety; (B) between the carboxyl group from Glu202 and H atom at NH group from indole moiety; (C) between O atom in OH group from Ser203 and H atom in NH group from indolinone; (D) between H atom in OH group from Ser203 and O atom in carbonyl group from indolinone; (E) between N atom from His447 and H1 atom in quaternary ammonium group; (F) between N atom from His447 and H2 atom in quaternary ammonium group; (G) between the positively charged N atom and Tyr337; (H) between Trp86 and indole part, and (I) between His447 and indolinone moiety. Figure S5. Distance plots over the course of simulation for compound **21**. (A) between carboxyl group from Asp74 and H atom in quaternary ammonium group; (B) between carboxyl group from Asp74 and positively charged N atom; (C) between H atom at OH group from Ser125 and O atom in CO group from the linker; (D) between indole moieties from Trp86 and 21; (E) between Tyr341 and benzene. Figure S6. Distance plots over the course of simulation for compound **9**. (A) between carboxyl group from Glu202 and H atom in quaternary ammonium group; (B) between carboxyl group from Glu202 and positively charged N atom; (C) between H atom at N-terminus from Gly121 and O atom at CO group from linker; (D) between H atom at N-terminus from Gly122 and O atom in CO group from linker; (E) between O atom in CO group from Tyr337 and H atom in amino group from the linker; (F) between the H atom at NH from His447 and O atom at the carbonyl group from the linker; (G) between indole moiety from Trp86 and cation in quaternary ammonium group. Figure S7. Distance plots over the course of simulation for compound **29**. (A) between H atom in OH group from Tyr133 and O atom in CO group; (B) between COO- from Glu202 and H atom in NH group from indole part; (C) between the indole moiety of Trp86 and positively charged ammonium atom. Figure S8. Distance plots over the course of simulation for GAL. (A) between H atom in NH group from Gly121 and O atom in OH group; (B) between COO- from Glu202 and H atom in NH group from indole part; (C) between the H atom from His447 and O atom in OH; (D) between O atom in OH from Tyr337 and H atom in NH; (E) between the benzene of Tyr124 and positively charged ammonium atom; (F) between the benzene of Tyr337 and positively charged ammonium atom; (G) between the indole moiety of Trp86 and the unsaturated double bond. Table S1: Natural compounds selected as potential AChE inhipitors by docking-based virtual screening and ADME filtering. Blue lines separate the different structural groups. The stable complexes from MD simulations are denoted in bold. The galantamine derivatives are presented in italics. Table S2. A three-step procedure of molecule selection for anti-AChE screening. ChemPLP scores from docking, averaged enthalpies with included solvent interactions, and sum of both absolute values are shown. The selected compounds are given in bold.

## Sample Availability

Samples of the compounds are available from the IBScreen.
