# Supplementary material for: Virtual Screening and Hit Selection of Natural Compounds as Acetylcholinesterase Inhibitors
Source: Molecules. 2022 May 13;27(10):3139. doi: 10.3390/molecules27103139 (PMC9145144; doi:10.3390/molecules27103139)
Supplement: Supplementary file 1 [file molecules-27-03139-s001.zip › molecules-1592786-supplementary.pdf]

Table S1. Natural compounds selected as potential AChE inhipitors by docking-based virtual screening and ADME filtering. Blue lines separate the different structural groups. The chosen suitable complexes from MD simulations are denoted in bold. The galantamine derivatives are presented in italic (**10** and **11**).

No	ZINC ID	ChemPLP	Structure	Size, Å	Vendors
**1**	**ZINC30726889**	**70.08**	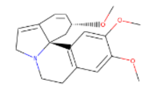	**5.755**	**YES**
**2**	**ZINC33955878**	**82.22**	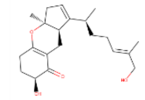	**5.704**	**YES**
3	ZINC15514750	83.6908	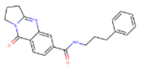	7.105	YES
4	ZINC12663453	81.385	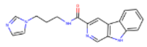	6.819	YES
**5***	**ZINC00488892**	**85.05**	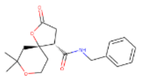	**5.155**	**YES**
6	ZINC00489733	75.6852	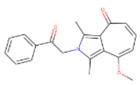	5.152	YES
**7**	**ZINC02161293**	**72.8523**	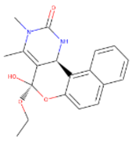	**7.456**	**YES**
8	ZINC04028616	80.7558	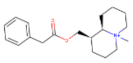	5.002	YES
**9***	**ZINC12296402**	**85.9159**	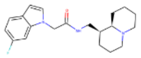	**5.1**	**YES**
10	*ZINC0402661*	*80.3642*	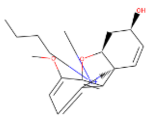	*6.265*	*YES*

No	ZINC ID	ChemPLP	Structure	Size, Å	Vendors
11	*ZINC96316410*	*71.611*	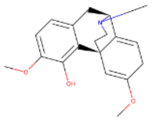	*5.27*	*YES*
12	ZINC04257807	73.9836	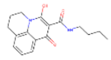	5.145	YES
13	ZINC19550899	75.1924	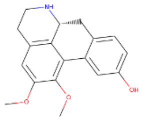	7.228	YES
14	ZINC19938823	77.898	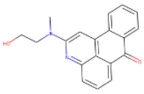	7.351	YES
15	ZINC04027315	73.6323	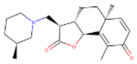	6.415	YES
**16***	**ZINC62001364**	**76.7413**	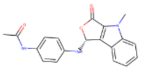	**6.491**	**YES**
**17***	**ZINC22607229**	**82.7631**	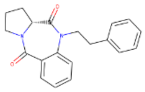	**7.311**	**YES**
**18***	**ZINC08764534**	**79.0189**	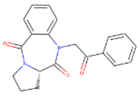	**7.323**	**YES**
**19**	**ZINC12296326**	**75.4476**	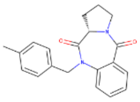	**7.152**	**YES**
**20**	**ZINC04027662**	**73.493**	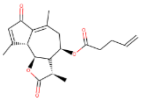	**5.698**	**YES**
**21***	**ZINC38571093**	**82.7599**	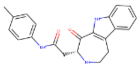	**7.4**	**YES**

No	ZINC ID	ChemPLP	Structure	Size, Å	Vendors
**22***	**ZINC06624539**	**84.6421**	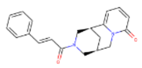	**5.33**	**YES**
**23**	**ZINC08764503**	**75.9211**	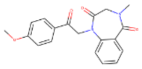	**5.087**	**YES**
24	ZINC12892572	80.7214	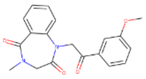	5.087	YES
**25***	**ZINC12902266**	**77.6389**	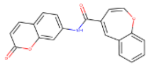	**5.184**	**YES**
**26**	**ZINC66407652**	**72.9183**	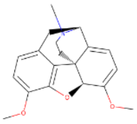	**5.588**	**YES**
27	ZINC12865585	71.3187	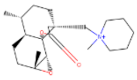	5.266	YES
**28***	**ZINC35877799**	**73.5909**	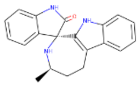	**8.735**	**YES**
**29***	**ZINC20064218**	**77.5505**	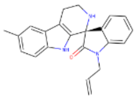	**7.421**	**YES**
30	ZINC36055840	72.3623	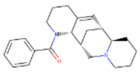	7.251	YES
**31**	**ZINC20410852**	**74.5599**	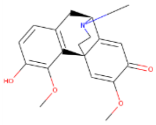	**6.503**	**YES**
**32**	**ZINC96316346**	**76.3434**	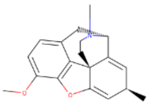	**6.123**	**YES**

No	ZINC ID	ChemPLP	Structure	Size, Å	Vendors
**33**	**ZINC96316411**	**78.0323**	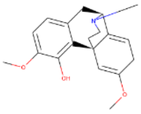	**6.565**	**YES**
**34**	**ZINC14719345**	**73.2384**	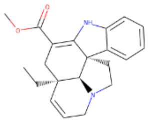	**5.919**	**YES**

* - selected compounds for testing

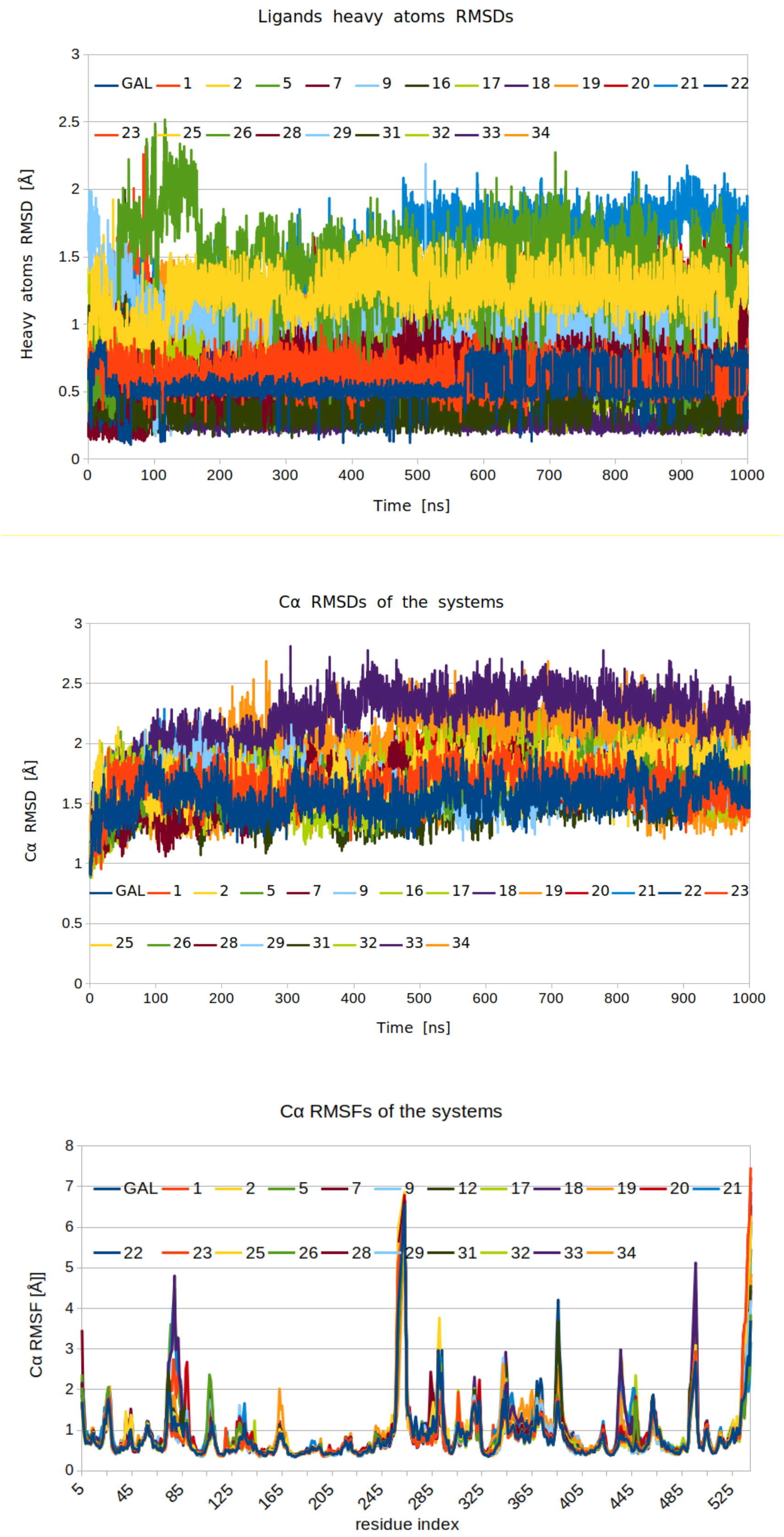

Figure S1. Ligands heavy atoms (up) and protein backbone C-atoms RMSDs (in the middle) and RMSFs (down) for AChE are shown.

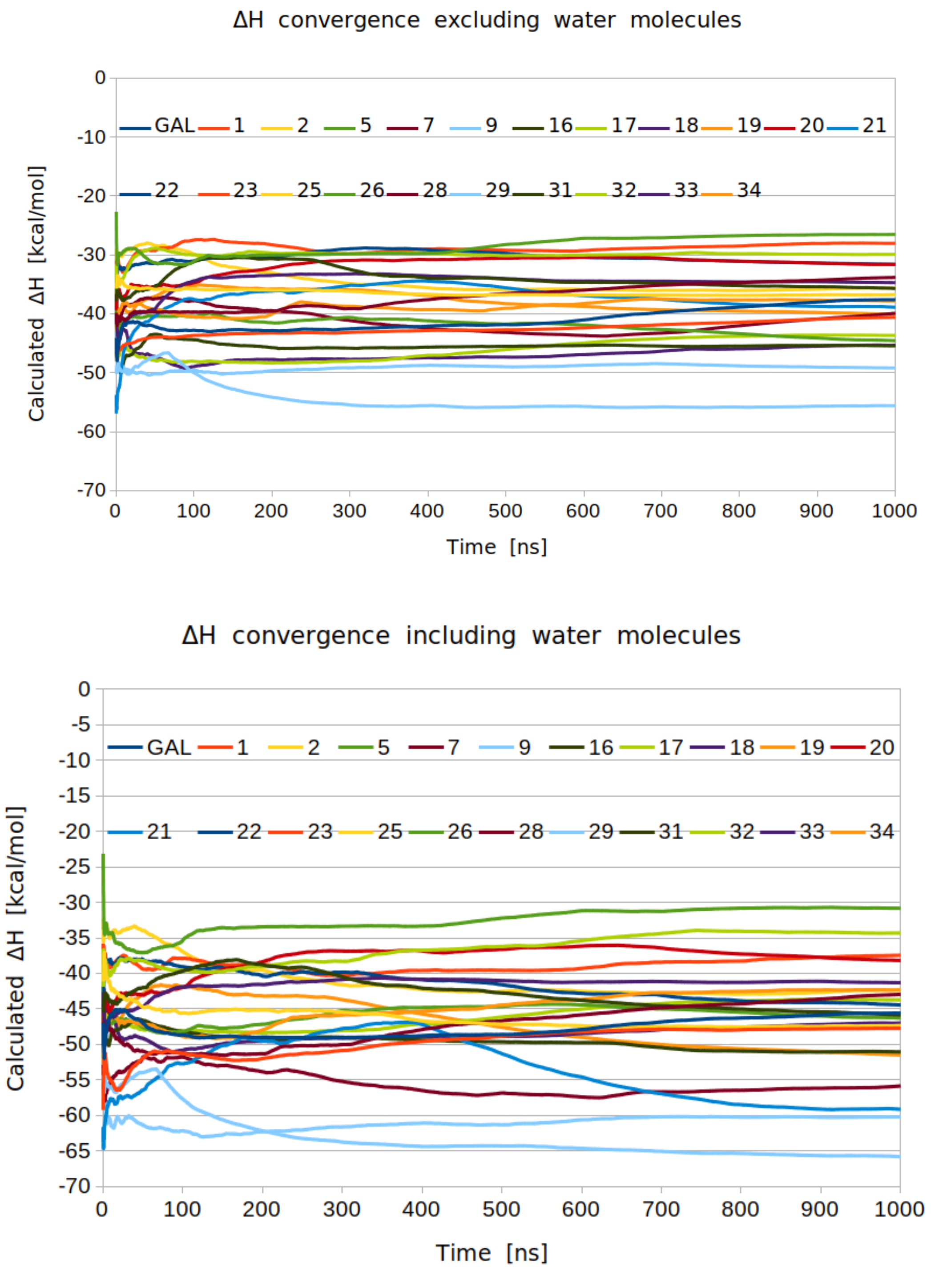

Figure S2. Convergence of enthalpies with excluded (up) and included (down) solvent interactions.

Table S2. A three-step procedure of molecule selection for anti-AChE screening. ChemPLP scores from docking, averaged enthalpies with included solvent interactions, and sum of both absolute values are shown. The selected compounds are given in bold.

Compound	ChemPLP score	$\Delta H_{solv,\ avrg}$, kcal/mol	ChemPLP+ $\lvert\Delta H_{solv,\ avrg}\rvert$	Price for 1mg, USD, InterBioScreen
1	70.08	-47.7340	117.8140	DV*
2	82.22	-47.3456	129.5656	>100
**5**	**85.05**	**-30.8296**	**115.8796**	**<100**
7	72.8523	-42.9445	115.7968	<100
**9**	**85.9159**	**-65.8063**	**151.7222**	**<100**
**16**	**76.7413**	**-45.8669**	**122.6082**	**<100**
**17**	**82.7631**	**-34.3182**	**117.0813**	**<100**
**18**	**79.0189**	**-41.3130**	**120.3319**	**<100**
19	75.4476	-42.3293	117.7769	<100
20	73.493	-38.1824	111.6754	<100
**21**	**82.7599**	**-59.1422**	**141.9021**	**<100**
**22**	**84.6421**	**-44.4386**	**129.0807**	**<100**
23	75.9211	-37.4531	113.3742	<100
**25**	**77.6389**	**-42.3505**	**119.9894**	**<100**
26	72.9183	-46.2868	119.2051	DV*
**28**	**73.5909**	**-55.8388**	**129.4297**	**<100**
**29**	**77.5505**	**-60.2032**	**137.7537**	**<100**
31	74.5599	-51.0576	125.6175	>100
32	76.3434	-43.7316	120.0750	DV*
33	78.0323	-47.0020	125.0343	DV*
34	73.2384	-51.4843	124.7227	>100
GAL				

*DV – different vendor

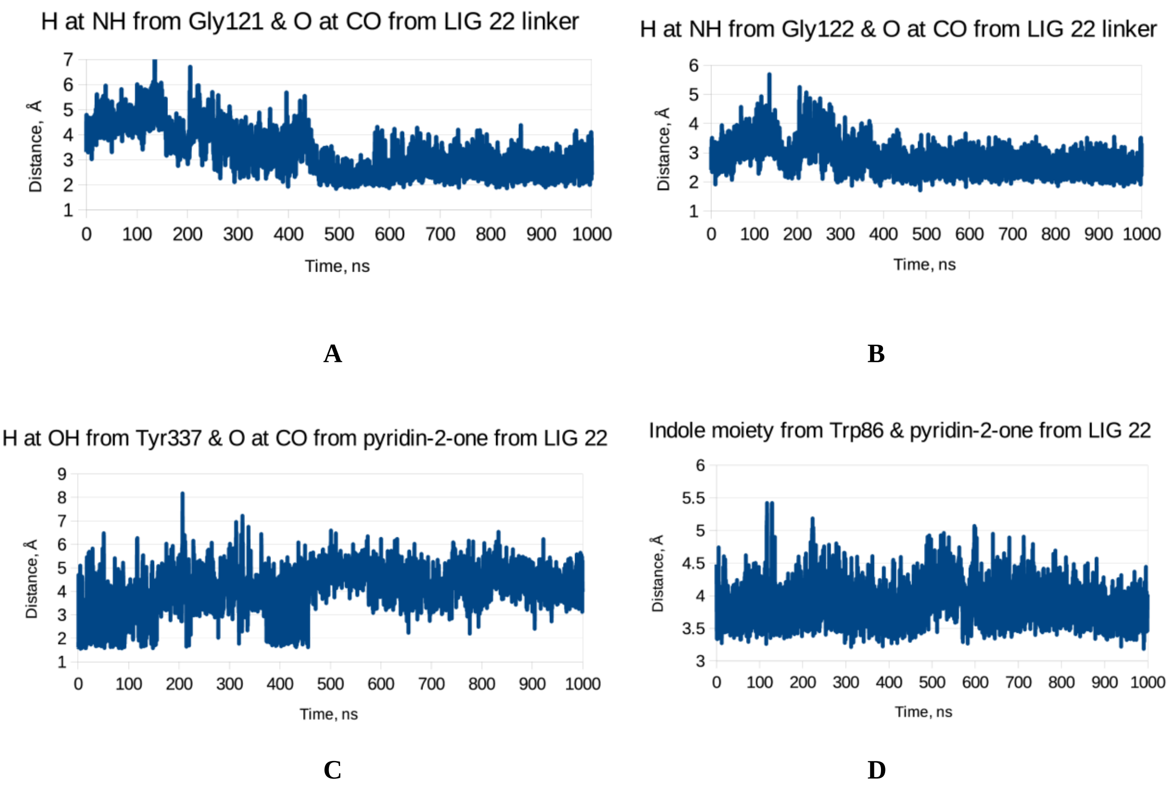

Figure S3. Distance plots over the course of simulation for compound **22**. A) between H atom at N-terminal from Gly121 and O atom at CO group from the linker; B) between H atom at N-terminal from Gly122 and O atom at CO group from the linker; C) between H atom at OH group from Tyr337 and O atom at CO group from pyridin-2-one ; D) between indole part of Trp86 and pyridin-2-one.

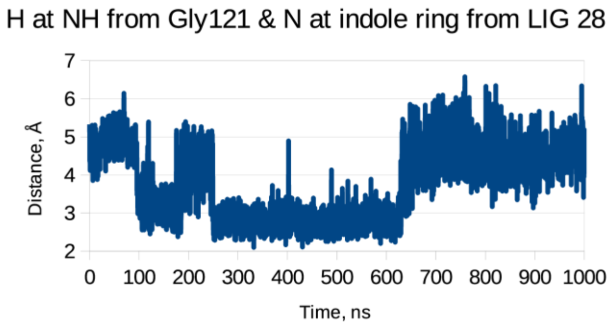
H at NH from Gly121 & N at indole ring from LIG 28
Distance, Å
Time, ns

A

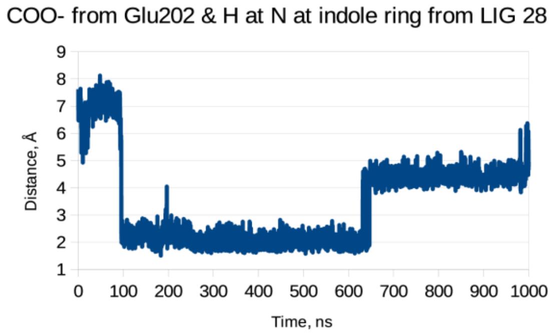
COO- from Glu202 & H at N at indole ring from LIG 28
Distance, Å
Time, ns

B

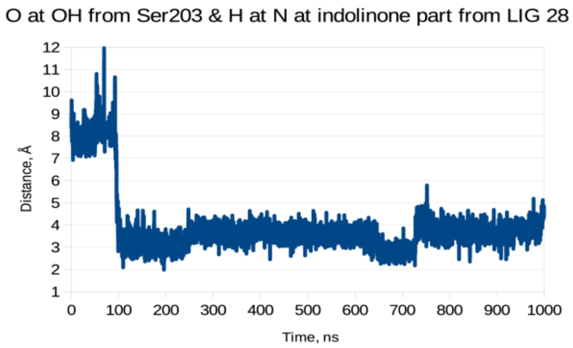
O at OH from Ser203 & H at N at indolinone part from LIG 28
Distance, Å
Time, ns

C

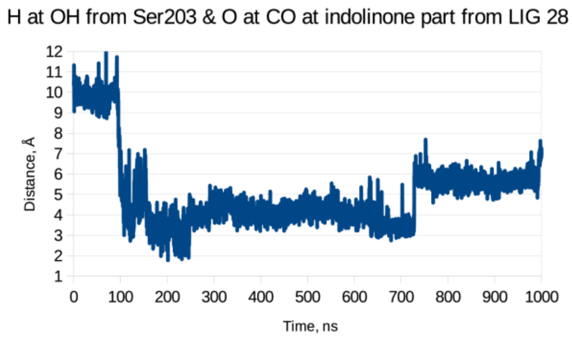
H at OH from Ser203 & O at CO at indolinone part from LIG 28
Distance, Å
Time, ns

D

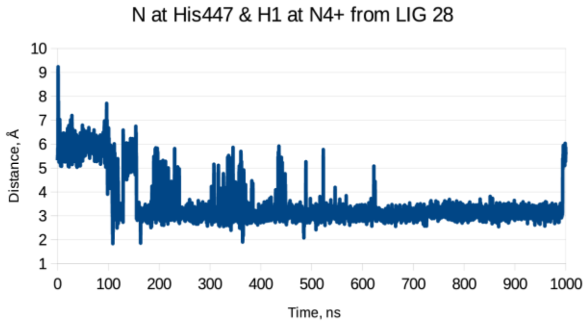
N at His447 & H1 at N4+ from LIG 28
Distance, Å
Time, ns

E

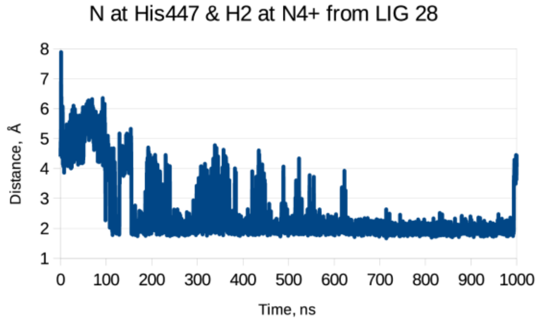
N at His447 & H2 at N4+ from LIG 28
Distance, Å
Time, ns

F

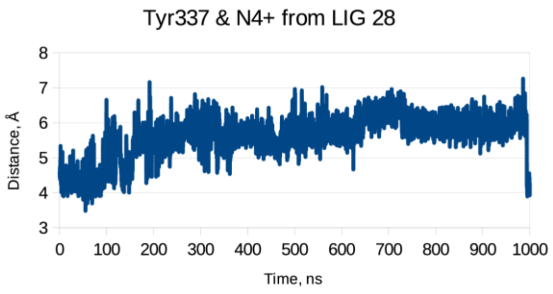
Tyr337 & N4+ from LIG 28
Distance, Å
Time, ns

G

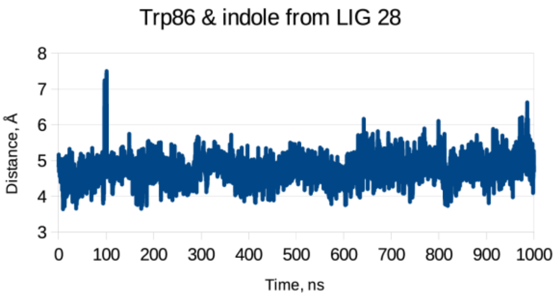
Trp86 & indole from LIG 28
Distance, Å
Time, ns

H

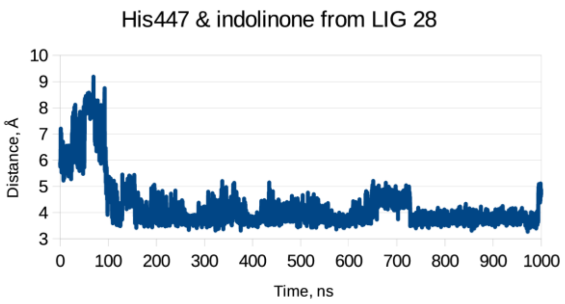

I

Figure S4. Distance plots over the course of simulation for compound **28**. A) between H atom at N-terminal from Gly121 and N atom from indole moiety; B) between the carboxyl group from Glu202 and H atom at NH group from indole moiety; C) between O atom at OH group from Ser203 and H atom at NH group from indolinone; D) between H atom at OH group from Ser203 and O atom at carbonyl group from indolinone; E) between N atom from His447 and H1 atom at quaternary ammonium group; F) between N atom from His447 and H2 atom at quaternary ammonium group; G) between the positively charged N atom and Tyr337; H) between Trp86 and indole part, and I) between His447 and indolinone moiety.

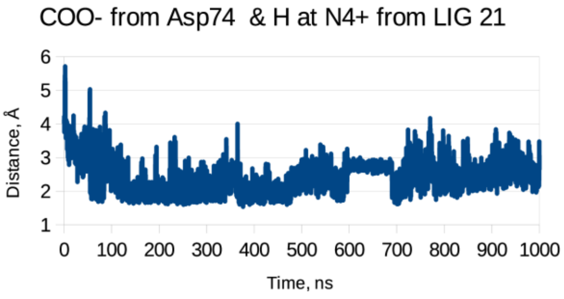

A

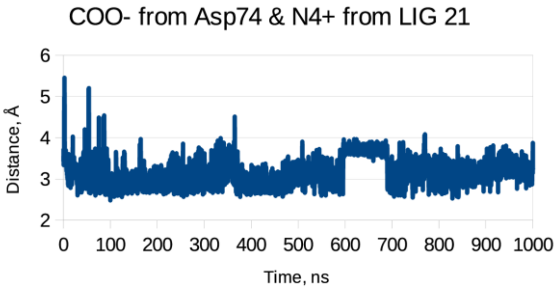

B

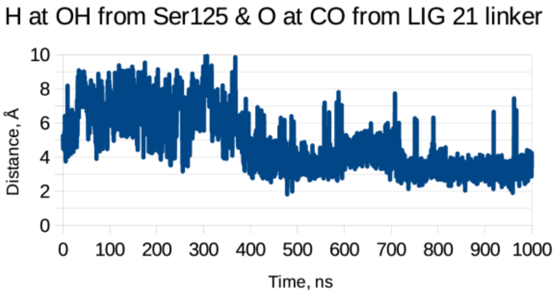

C

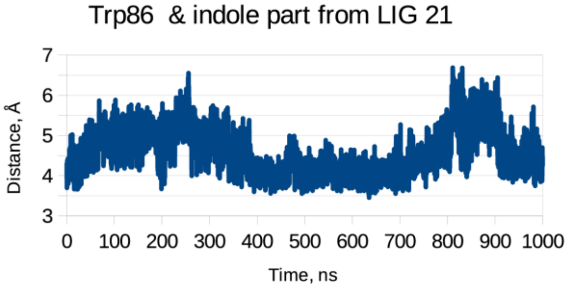

D

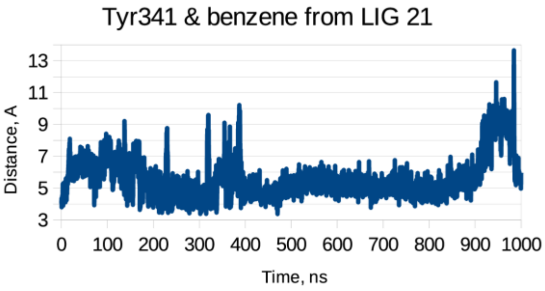

E

Figure S5. Distance plots over the course of simulation for compound **21**. A) between carboxyl group from Asp74 and H atom at quaternary ammonium group; B) between carboxyl group from Asp74 and positively charged N atom; C) between H atom at OH group from Ser125 and O atom at CO group from the linker; D) between indole moieties from Trp86 and **21**; E) between Tyr341 and benzene.

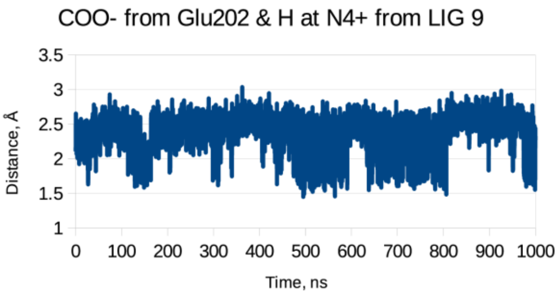
COO- from Glu202 & H at N4+ from LIG 9
Distance, Å
Time, ns
3.5
3
2.5
2
1.5
1
0 100 200 300 400 500 600 700 800 900 1000

A

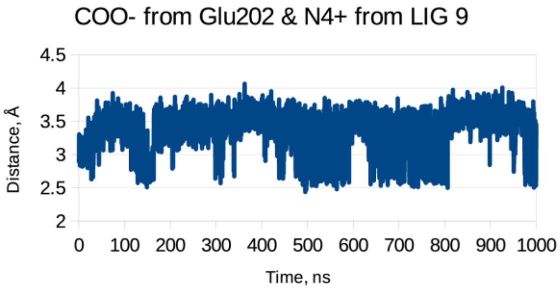
COO- from Glu202 & N4+ from LIG 9
Distance, Å
Time, ns
4.5
4
3.5
3
2.5
2
0 100 200 300 400 500 600 700 800 900 1000

B

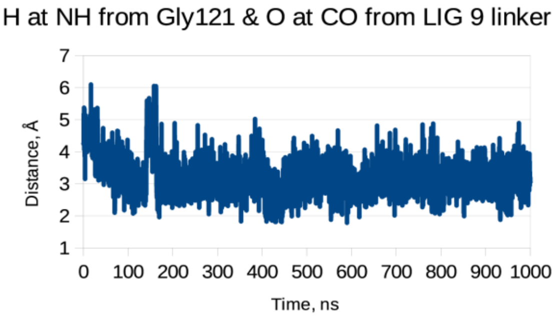
H at NH from Gly121 & O at CO from LIG 9 linker
Distance, Å
Time, ns
7
6
5
4
3
2
1
0 100 200 300 400 500 600 700 800 900 1000

C

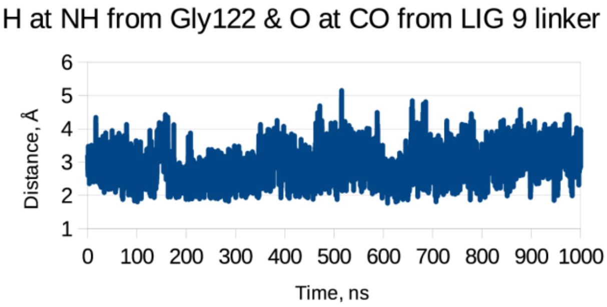
H at NH from Gly122 & O at CO from LIG 9 linker
Distance, Å
Time, ns
6
5
4
3
2
1
0 100 200 300 400 500 600 700 800 900 1000

D

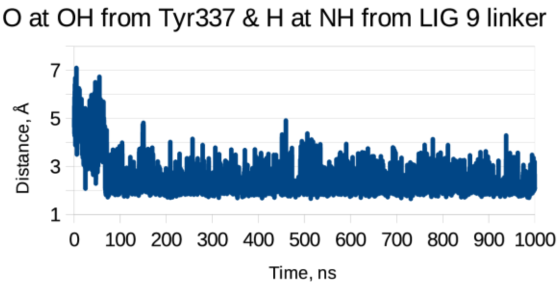
O at OH from Tyr337 & H at NH from LIG 9 linker
Distance, Å
Time, ns
7
5
3
1
0 100 200 300 400 500 600 700 800 900 1000

E

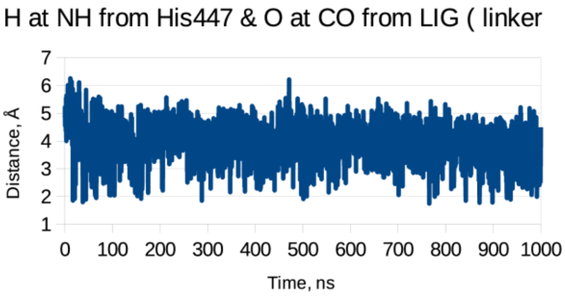
H at NH from His447 & O at CO from LIG (linker
Distance, Å
Time, ns
7
6
5
4
3
2
1
0 100 200 300 400 500 600 700 800 900 1000

F

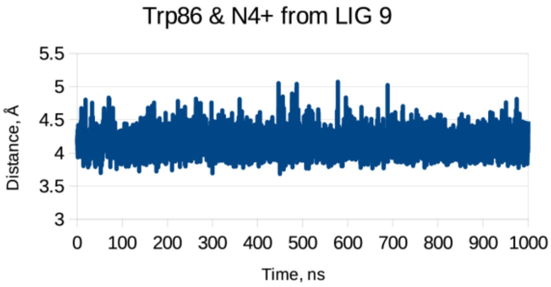
Trp86 & N4+ from LIG 9
Distance, Å
Time, ns
5.5
5
4.5
4
3.5
3
0 100 200 300 400 500 600 700 800 900 1000

G

Figure S6. Distance plots over the course of simulation for compound **9**. A) between carboxyl group from Glu202 and H atom at quaternary ammonium group; B) between carboxyl group from Glu202 and positively charged N atom; C) between H atom at N-terminus from Gly121 and O atom at CO group from linker; D) between H atom at N-terminus from Gly122 and O atom at CO group from linker; E) between O atom at CO group from Tyr337 and H atom at amino group from the linker; F) between the H atom at NH from His447 and O atom at the carbonyl group from the linker; G) between indole moiety from Trp86 and cation at quaternary ammonium group.

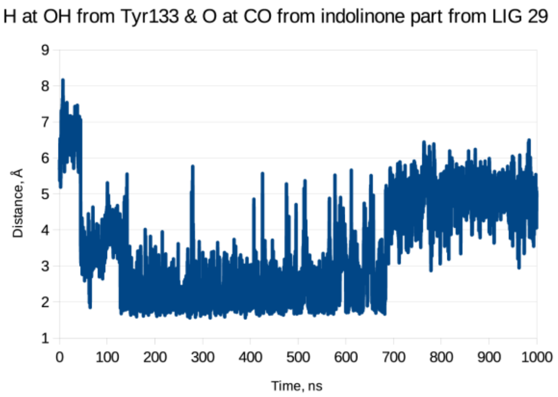

A

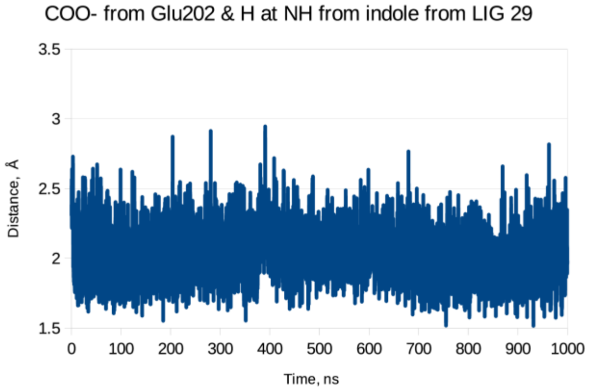

B

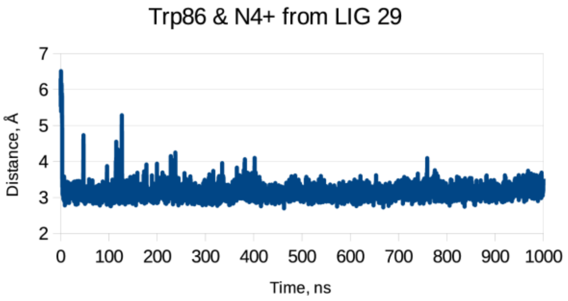

C

Figure S7. Distance plots over the course of simulation for compound **29**. A) between H atom at OH group from Tyr133 and O atom at CO group; B) between COO^- from Glu202 and H atom at NH group from indole part; C) between the indole moiety of Trp86 and positively charged ammonium atom.

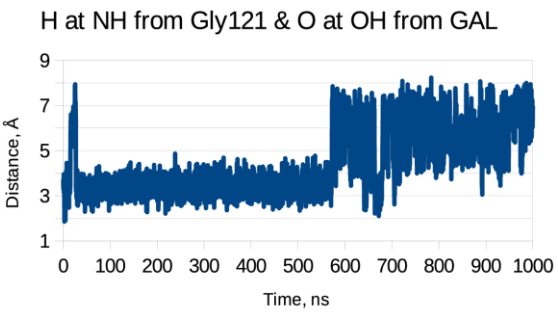
H at NH from Gly121 & O at OH from GAL
Distance, Å
Time, ns

A

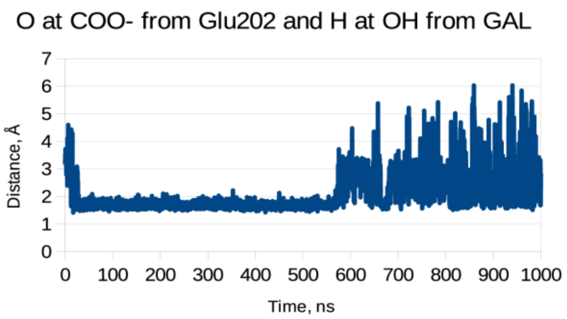
O at COO- from Glu202 and H at OH from GAL
Distance, Å
Time, ns

B

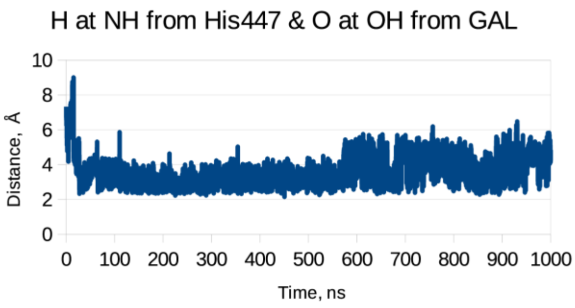
H at NH from His447 & O at OH from GAL
Distance, Å
Time, ns

C

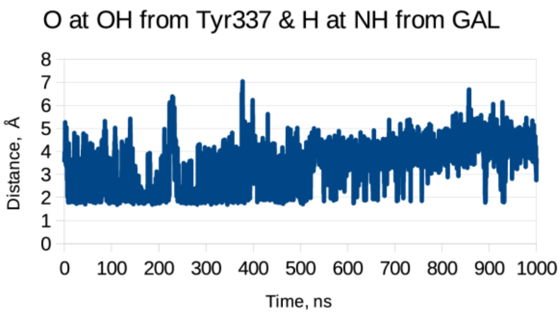
O at OH from Tyr337 & H at NH from GAL
Distance, Å
Time, ns

D

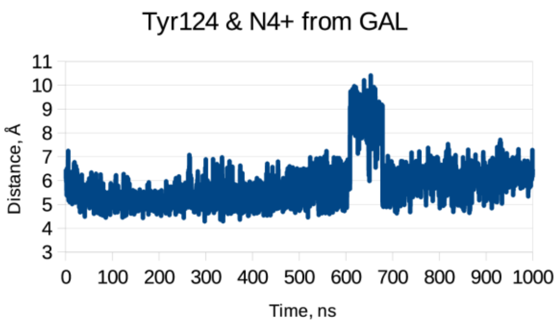
Tyr124 & N4+ from GAL
Distance, Å
Time, ns

E

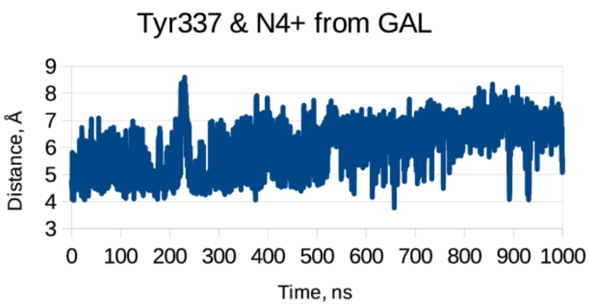
Tyr337 & N4+ from GAL
Distance, Å
Time, ns

F

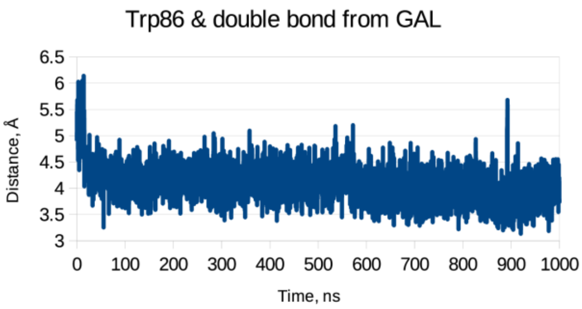
Trp86 & double bond from GAL
Distance, Å
Time, ns

G

Figure S8. Distance plots over the course of simulation for GAL. A) between H atom at NH group from Gly121 and O atom at OH group; B) between COO^- from Glu202 and H atom at NH group from indole part; C) between the H atom from His447 and O atom at OH; D) between O atom at OH from Tyr337 and H atom at NH; E) between the benzene of Tyr124 and positively charged ammonium atom; F) between the benzene of Tyr337 and positively charged ammonium atom; G) between the indole moiety of Trp86 and the unsaturated double bond.
